# Centrosome Dysfunction Contributes to Chromosome Instability, Chromoanagenesis, and Genome Reprograming in Cancer

**DOI:** 10.3389/fonc.2013.00277

**Published:** 2013-11-12

**Authors:** German A. Pihan

**Affiliations:** ^1^Department of Pathology and Laboratory Medicine, Beth Israel Deaconess Medical Center, Harvard Medical School, Boston, MA, USA

**Keywords:** aneuploidy, centrosome, centriole, chromoanagenesis, chromothripsis, micronuclei, mitosis, CIN

## Abstract

The unique ability of centrosomes to nucleate and organize microtubules makes them unrivaled conductors of important interphase processes, such as intracellular payload traffic, cell polarity, cell locomotion, and organization of the immunologic synapse. But it is in mitosis that centrosomes loom large, for they orchestrate, with clockmaker’s precision, the assembly and functioning of the mitotic spindle, ensuring the equal partitioning of the replicated genome into daughter cells. Centrosome dysfunction is inextricably linked to aneuploidy and chromosome instability, both hallmarks of cancer cells. Several aspects of centrosome function in normal and cancer cells have been molecularly characterized during the last two decades, greatly enhancing our mechanistic understanding of this tiny organelle. Whether centrosome defects alone can cause cancer, remains unanswered. Until recently, the aggregate of the evidence had suggested that centrosome dysfunction, by deregulating the fidelity of chromosome segregation, promotes and accelerates the characteristic Darwinian evolution of the cancer genome enabled by increased mutational load and/or decreased DNA repair. Very recent experimental work has shown that missegregated chromosomes resulting from centrosome dysfunction may experience extensive DNA damage, suggesting additional dimensions to the role of centrosomes in cancer. Centrosome dysfunction is particularly prevalent in tumors in which the genome has undergone extensive structural rearrangements and chromosome domain reshuffling. Ongoing gene reshuffling reprograms the genome for continuous growth, survival, and evasion of the immune system. Manipulation of molecular networks controlling centrosome function may soon become a viable target for specific therapeutic intervention in cancer, particularly since normal cells, which lack centrosome alterations, may be spared the toxicity of such therapies.

Cancer is an evolutionary multistep process arising in single cells resulting from accumulation of non-lethal mutations that increase, decrease, deregulate, or interfere with the function of critical genes, leading to autonomous growth and loss of homeostasis. Cancer cells fail to execute programed cell death when required, fail to exit the cell cycle when prompted, or to differentiate in response to appropriate external or internal regulatory signals. This dynamic “renegade” behavior (1), which was elegantly codified by Hanahan and Weinberg in a series of publications addressing the Hallmarks of cancer cells (2, 3), lay at the core of cancer biology and dispels the notion of sporadic cancer as a simple, oligogenic somatic genetic disease. Cancer is indeed a family of complex evolutionary somatic genetic disorders resulting from dynamic and ongoing reprograming of the genome, the nature of which will continue to challenge our ingenuity for years to come.

## The First Cancer Cell Hallmark

**Figure 1:**
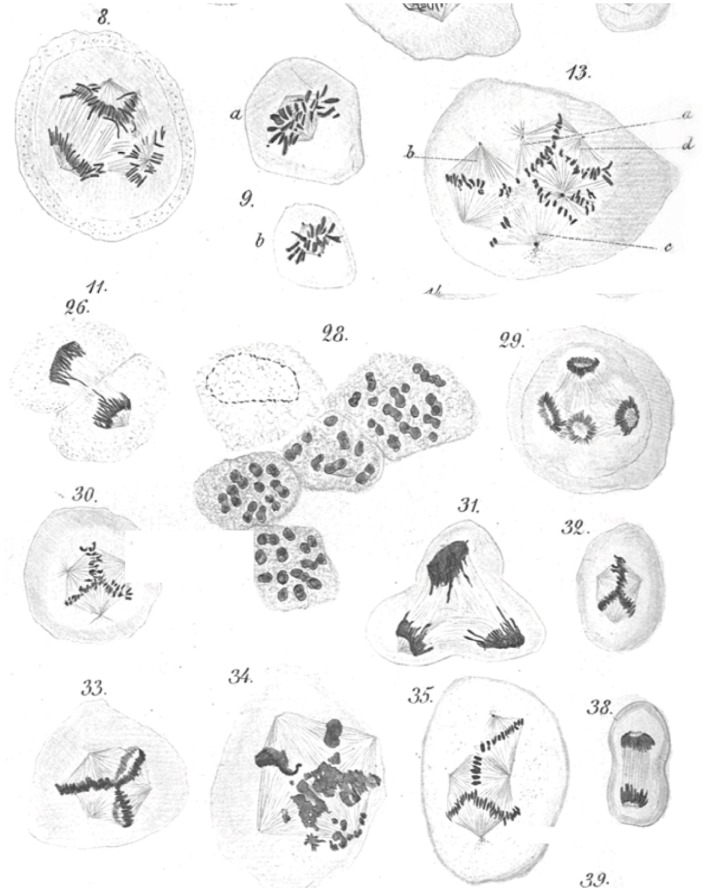
**Leo Hansemann’s drawings of abnormal mitoses in cancer tissue**. Abnormal metaphases (13, 30, 32, 33, 35), anaphases (8, 29), and telophases (31) are exquisitely represented, with many of the drawings implying supernumerary centrosomes, which could not be directly visualized in these preparations. Apparent mitotic catastrophes are represented as well (28, 34) (reproduced with permission from Virchows Archive). Hansemann took care of placing cancer tissue samples in warm fixative immediately after surgical resection to avoid anoxia-induced changes and in the process beautifully preserved spindle microtubules.

An abnormal complement of chromosomes, i.e., aneuploidy, is arguably the first identified hallmark of cancer cells. Beginning in 1890 Leo Hansemann, in a series of beautifully illustrated observations (Figure 1), documented the frequent presence of asymmetric and multipolar mitoses in carcinoma tissue (4, 5). Though uncertain of the significance of his findings, Hansemann was aware that daughter cells resulting from asymmetric mitoses received abnormal amounts of “chromatin.” Perhaps in part prompted by these findings, Theodore Boveri, who was aware of Leo Hansemann’s work and publically acknowledged his findings, formulated his now famous theory of cancer development (6). Half a century later, Torbjörn Caspersson, who pioneered cytological microspectrofluorimetric analysis of nuclei acids (7), would resoundingly confirm Hansemann and Boveri’s predictions. Caspersson was the first to observe that cancer cells, unlike normal cells, which always contained a constant amount of DNA, almost always exhibited greater, but highly variable quantities of nuclear DNA (8). It can thus be stated that an abnormal chromosome complement, possibly resulting from abnormal centrosome function, was the first hallmark of cancer ever identified. Since these pioneering observations, the nearly universal occurrence of abnormal chromosomes in cancer, in a bewildering combination of numerical and structural abnormalities, has been widely documented. A number of data repositories, such as The Cancer Genome Anatomy Project (9, 10), which includes Mitelman’s cancer cytogenetic collection currently containing 62,601 cancer karyotypes (11), provide a plethora of data and an overview of the spectrum and extent of large scale genome changes in cancer.

## Contemporary View on Centrosomes Structure and Function

**Figure 2:**
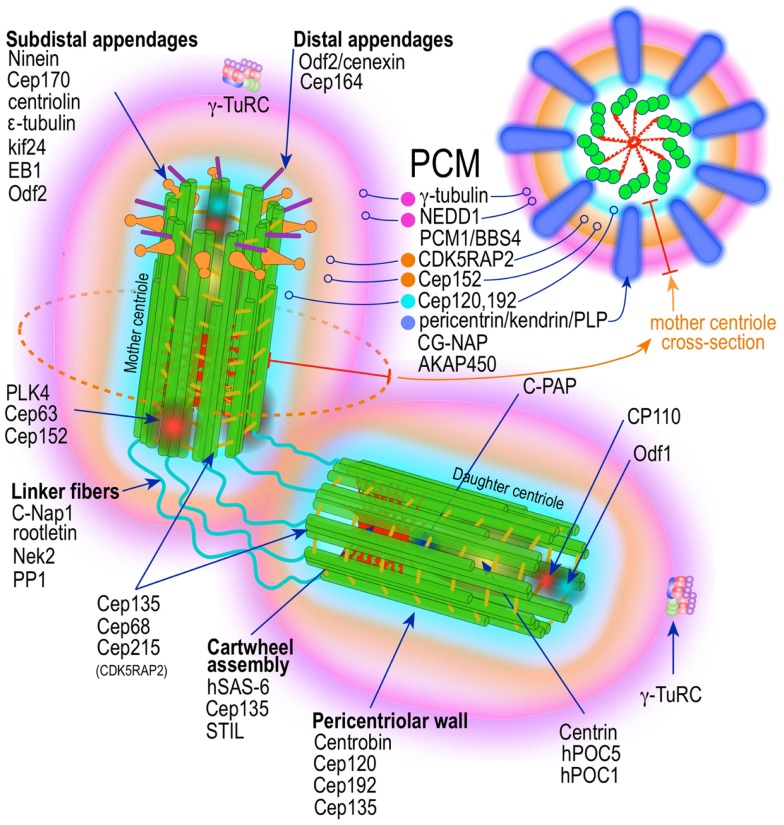
**Prototypic vertebrate centrosome**. Salient architectural features of the post-mitotic centrosome (after centriole disengagement, but before procentriole nucleation) include fully developed mother and daughter centrioles and electron dense but highly structured pericentriolar material (PCM).

The centrosome is a multifunctional structurally complex macromolecular machine composed of hundreds of proteins (12–16) (Figure 2). It is the primary microtubule-organizing center (MTOC) in metazoans (17–26) controlling several interphase and mitotic microtubule-dependent processes (17–19, 22–25, 27).

In interphase, the centrosome establishes and controls the microtubule network that serves as the highway for fast intracellular payload traffic of protein and organelles (26). It contributes to the development and maintenance of cell polarity (28), cell adhesion (29), cell locomotion (30), and the organization of the immunological synapse (31). In differentiated cells the maternal centriole guides the establishment of the primary cilium (32). However, it is during mitosis that centrosomes play their most visible role. Centrosomes organize and fine-tune the microtubule arrays that form the mitotic spindle – the elegant and precisely choreographed supramolecular machine that ensures the segregation of exactly one full diploid set of chromosomes to each daughter cell during mitosis (21, 33). Additionally, astral microtubules arising in centrosomes control mitotic spindle position and orientation (34–36), thereby specifying the cell division plane, which ultimately control cell fate in some stem cell niches, such as the developing central nervous system (34–36). The centrosome, specifically its maternal centriole, is critical for the development of the primary and secondary cilium in differentiated cells.

The centrosome possesses microtubule-independent functions as well. The most important of these is its cell cycle regulatory activity. Several proteins that localize to centrosomes sequentially control a number of cell cycle processes. Centrosomes license post-mitotic G1 cells for entry into the next S phase (37). In addition, centrosomes may control the timing of S-phase and mitotic entry. Cyclins A and E have centrosome localization sequences (CLS) which are critical for Cdk2-dependent S-phase entry. Interference with cycle A/E binding to the centrosome prevents entry into S phase (38). Centrosomal localization of Cyclin E-Cdk2 is required for initiation of DNA synthesis (39), whereas Cyclin A-Cdk2 by binding MCM5 and Orc1 – two proteins involved in DNA replication – prevents centrosome overduplication in S phase (40). Centrosomes may also control the initial activation of cyclin B1-Cdk1 in the cytoplasm, which is critical for mitotic entry. Enzymatically active cyclin B1-Cdk1 appears first at the centrosome at the onset of mitosis (41, 42). Cyclin B-Cdk1 is maintained inactive via phosphorylation of ATP binding pocket amino acids Thr14 and Tyr15 by Wee kinase. Mitotic onset is triggered by a massive wave of Cyclin B-Cdk1 activation that starts at the centrosome by Cdc25B phosphatase initially dephosphorylating Cdk1. This initially activated centrosomal pool of Cyclin B-Cdk1 at the end of prophase, phosphorylates Cdc25B and Wee1, activating the first and inhibiting the second, and triggering a rapidly expanding wave of Cyclin B-Cdk1 activation with its epicenter at the centrosome. The importance of preventing activation of the centrosomal pool of Cyclin B-Cdk1 is highlighted by it being closely guarded by the checkpoint kinase Chk-1, which localizes to the centrosome in interphase, but not mitosis, and inhibits Cdk1 activation (43). Inhibition of centrosomal Chk-1 results in premature separation of centrosomes and activation of Cyclin B-Cdk1 leading to premature entry into mitosis (44). Moreover, Aurora kinase A – which localized to the centrosome and increases through S and G2 phases – appears to be the trigger for nuclear envelope breakdown (NEB) in mitosis (45). Cyclin F, the only F-box cyclin, accumulates in the centrosome in G2 and is involved in ubiquitinylation of CP110 targeting it for proteolytic degradation. Degradation of CP110 ensures restriction of centrosome replication to once in each cell cycle. Cyclin F also targets the ribonucleotide reductase RRM2, leading to downregulation of dNTP pools at the end of S phase (46). Proteolytic cleavage of CP110 and RRM2 are important to prevent genomic instability. Finally, in some cells the maternal centrosome, late during cytokinesis, moves close to the central spindle and appears to license abscission by releasing central spindle microtubules (47). This function appears to enhance the fidelity of cytokinesis (48). Considered together, these findings reveal that the centrosome is an integral component and relay station for signals that control events throughout the cell cycle, functions that if disrupted may lead to abnormal DNA replication and cell division.

### Centrosome integrity checkpoint

In the course of experiments investigating the function of several centrosome components, an important aspect of centrosome self-regulation was revealed. Depletion of centrosomal AKAP450 and pericentrin, by targeting their common centrosome binding domains induces post-mitotic cell cycle arrest. Moreover, targeted depletion of 14 out of 15 centrosome components by siRNA, prolonged cytokinesis and triggered G1 arrest in telomerized (immortal), non-transformed human cells (49, 50). Cell cycle arrest occurred from within G1, was independent of mitosis and cytokinesis, and dependent on p38-induced phosphorylation of p53 at Ser-33. Ser-33 phosphorylated p53 localizes to the centrosome from which it translocates into the nucleus upregulating p21 and inducing S1 arrest via inhibition of Cdk2-CyclinA/E activity (50). This seemingly universal G1 arrest response of non-transformed cells to centrosome “injury” constitutes a *de facto* “centrosome integrity checkpoint” that precludes normal cells from reentering the cell cycle once centrosome integrity has been compromised, thus avoiding chromosome instability (CIN) and genomic instability (50).

## Centrosome Structure

### Centrioles

A mature centrosome is comprised of two centrioles in orthogonal configuration, surrounded by an amorphous electron-dense protein-rich cloud, termed pericentriolar material (PCM) (51–53) (Figure 2). Each centriole is a cylindrical structure made up of nine parallel microtubule triplets, their long axes running the length of the cylinder. Each microtubule triplet is composed of an internal A, middle B, and external C microtubule. Unlike A and B, the C microtubule runs only two-thirds the length of the centriole and is absent from its distal portion. In the proximal centriole, the A microtubule of one triplet is connected to the C microtubule of the previous triplet by an A-C linker, which becomes an A-B linker beyond the end of the C microtubule toward the distal end of the centriole barrel. The microtubule triplets are heavily acetylated (54) and polyglutamylated (55) and thus very stable, exchanging little if any with the soluble pool of αβ tubulin heterodimers. The ninefold radial symmetry of centrioles is apparently determined and maintained by the proximal-end cartwheel assembly (Figure 2). Comprised of nine spokes radiating from a central hub, the cartwheel assembly is composed almost entirely of a single protein, Sas-6. The cartwheel assembly is repeated six times along the interior of the proximal end of the centriole. Although nearly identical, the older, “mother” centriole, additionally exhibits distal and subdistal appendages, which are essential for centrosome tethering to the plasma membrane and initiation of ciliogenesis (56). In G1 phase of the cell cycle, disengaged mother and daughter centrioles are tethered to each other by intercentriolar fibers. Attesting to the complexity of this organelle, many structural and regulatory proteins have specific locations within the centriole (Figure 2).

### Pericentriolar material

While centrioles are essential to restrict centrosome duplication to once in every cell cycle (57, 58), they are largely dispensable for microtubule nucleation. It is instead the pericentriolar amorphous cloud of proteins, the PCM, the structure responsible for most of the centrosome microtubule-organizing activity, including microtubule nucleation and control of microtubule number, polarity, distribution, and flux (21) (Figure 2). Until both centrioles are fully mature at the end of G2, most of the PCM is associated with the mother centriole (58, 59). This ensures that the developing daughter centriole in S phase remains “inactive.” Of the hundreds of proteins that localize to the pericentriolar cloud, γ-tubulin plays a central role by providing a template for the initiation of polymerization of α- and β-tubulin heterodimers into growing microtubules. γ-tubulin (TUBG1, TUBG2) performs this task in complex with other proteins collectively known as gamma tubulin complex proteins (GCP2–6 or TUBGCPs) (60–62). GCPs exhibit conserved “grip domains” at both N- and C-terminal ends (63). Two copies of γ-tubulin (GCP1) together with GPC2 and GPC3 form a tetramer termed γ-tubulin small complex (γ-TuSC) (64, 65). Several γ-TuSC, together with GCPs 4–6, assemble into a higher-order complex with a toroidal shape known as γ-tubulin ring complex (γTuRC) (62, 65–69) (Figure 2). The γTuRC toroid is stabilized on one of its sides by a complex of GCPs 4–6 proteins (70). Two other core components of the γTuRC complex, which do not contain Grip domains, GCP-WD (GCP7, NEDD1) and GCP8 (MOZART2), are non-essential for γTuRC assembly (71–74). However, GCP-WD is essential for γTuRC attachment to the PCM. The C-terminus of GCP-WD binds γ-tubulin in the γTuRC and the N-terminus WD domains form the blades of a β-propeller structure that binds to the PCM (72, 73, 75–77). GCP8 (MOZART2) appears to play a role in the recruitment of γTuRC to the PCM during interphase (74). An additional γTuRC core component is GCP9 (MOZART1) (74, 78). In human cells MOZART1 is required for recruitment of γTuRC to mitotic centrosomes (78). While Grip-GCPs and γ-tubulin are considered structural components of the γTuRC (72, 73, 75–77), some GCPs may have regulatory functions as well. γTuRC is targeted to MTOCs with the help of several centrosomal proteins, including AKAP450 (CG-NAP, AKAP9) (79), pericentrin (PCNT) (80), and CDK5RAP2 (Cep215) (81), and in human cells is dependent on an intact mature γTuRC (82). However, attachment of γTuRC to the centrosome occurs via GCP-DW (NEDD1) and this attachment factor is recruited to the centrosome independently of the γTuRC (72, 73). A number of non-stoichiometric regulatory molecules preferentially associate with the γTuRC in mitosis (72, 73, 78). These include Plk1, and seven of the eight Augmin/HAUS complex subunits (78) (Figure 2). The Augmin complex was initially defined in *Drosophila* as an eight-subunit centrosome protein complex of 340 kDa that localizes γTuRC to the mitotic spindle where it is indispensable for nucleating microtubules in a centrosome-independent manner, increasing microtubule density within the spindle, and stabilizing kinetochore microtubules (83). Humans possess a similar protein complex termed HAUS1–8 (78, 83, 84). Just as Augmin, the HAUS complex resides in the centrosome PCM and moves to spindle microtubules during mitosis, where it is important to increase the density of kinetochore and polar microtubules (83). In the absence of HAUS, mitotic human cells have reduced spindle tension (insufficient number of microtubules) and are unable to extinguish the mitotic spindle assembly checkpoint, leading to stalled mitosis and microtubule-dependent centrosome fragmentation (84). HAUS1–8 complexes also have a role in cytokinesis (83). The function of the individual HAUS units is currently poorly understood. It is thought that HAUS8 directly contact microtubules, while HAUS6 binds γTuRC via NEDD1 (83).

Recently, superresolution microscopy has begun to provide insights into the organization of the PCM (85–87). With the exception of an early study using fluorescence microscopy with image deconvolution that suggested the PCM is organized in a lattice-like structure around the centriole (88), it has been generally believed that the PCM is largely unstructured. Using SIM and STORM, two forms of superresolution fluorescence microscopy, Mennella et al (87) have demonstrated that the PCM in *Drosophila* S2 cells, contrary to the prevailing view, is highly organized into two or three main structural domains (Figure 2). One layer juxtaposed to the centriole wall and a second, matrix-like layer, located further away. Some coil–coil proteins in the juxtacentriolar layer, such as pericentrin-like protein (PLP), have their carboxy termini located near the centriole wall from which they extend, in clusters with quasi ninefold symmetry, centrifugally into the matrix layer. RNA interference experiments indicate that the juxtacentriolar layer is fundamentally required for organization of the external matrix layer. Remarkably, many of the proteins of the PCM have distinctive and strictly defined distribution volumes around the maternal centriole. With Sas-6 located at the center of the centriole and exhibiting the smallest distribution volume, followed by Sas-4, known to localize to the centriole wall, followed by PLP and asterless (ASL) in the inner region and SPD-2, γ-tubulin, and CNN in the outer matrix-like region further away from the centriole (87). Until mitosis PLP distributes around the mother centriole only. Beginning in mitosis PLP also distributes to the daughter centriole, a process that is completed by the end of telophase. In mammalian cells, pericentrin/kendrin and CG-NAP, two PACT (pericentrin-AKAP450 centrosome targeting) proteins, have a distribution similar to *Drosophila* PLP, which is consistent with PCNT/Kendrin being the ortholog of PLP. PCNT associates with CEP192 (SPD-2) and CDK5Rap2 in a functional complex in the inner layer of the PCM (89–93) (Figure 2). It is apparent that this layered organization of the PCM subserves two different needs, the inner layer as an organizer of the PCM and the outer layer with most of the microtubule nucleation functionality by providing docking sites for the γTuRC (87) (Figure 2). A similar study using mammalian cells revealed essentially equivalent organization of the PCM (86). Again PCNT distribution and orientation was consistent with its role in organizing the external PCM toroid responsible for microtubule nucleation (86). γ-tubulin and NEDD1 distributed to a region estimated to be at the center of the partially extended PCNT (Figure 2). Nevertheless, the distributions of components of the PCM seem to be even more complex that these initial studies revealed. A large survey of centrosome/centriole protein localization in interphase U2OS cells using 3D-SIM revealed that γ-tubulin and NEDD/GCP-WD, in addition to their distribution around the mother centriole and to a lesser extent around the daughter centriole, are also present, in a dot-like pattern, inside the mother centriole, as the gravitational center of the pericentriolar ring (94). Remarkably, this is consistent with previous immuno-EM data demonstrating these two proteins also residing inside the centriole (95, 96). Examination of the distribution, orthogonal to the mother centriole long axis, of an additional 18 proteins revealed distinctive pattern consisting of either toroid-like or dot-like structures. Four proteins (centrin, Sas-6, STIL, and Plk-4) distributed in compact dots. All other proteins (94) revealed toroid structures of varying sizes. Only one other protein, CPAP showed both toroid and central dot-like localization. Of the toroid proteins Cep135 and CP110 formed rings of nearly identical size, but distributed to the opposite ends (proximal and distal, respectively) of the centriole, in agreement with previous immuno-EM data (97, 98). All other proteins formed toroids of larger diameter, which were separated into three arbitrary groups. The inner group is composed by Cep192, NEDD1, Cep152-C, Cap350; the intermediate by Cep152-N, Cep215, γ-tubulin; and the outer layer by CPAP and pericentrin. Cep164 localized to the distal appendages, whereas Cep170 and ninein to subdistal appendages. These data indicate the γTuRC distributes between the inner and intermediate layers of the PCM in interphase U2OS cells. By using amino- or carboxy-terminal specific antibodies, and other domain specific antibodies, it was possible to demonstrate that some of these protein have not only specific localization, but also defined orientations within the PCM (94). The proteins with the largest distribution volumes were ninein (99) and Cep170 (100), which localized to the centriole subdistal appendages, whereas the distal appendage protein Cep164 (101) had slightly lower distribution volume. The location of these proteins is concordant with their previous localization by electron microscopy (52). CP110 and Centrin localizes to the distal end of centrioles (97, 98).

These studies combined indicate that the PCM, in spite of its amorphous appearance under the regular fluorescence or electron microscope, is highly organized (Figure 2). In cells in interphase it is distributed onto roughly two pericentriolar cylinders. An inner layer closely apposed to centriolar microtubules, composed of Sas-4, Spd-2, and Polo kinase, and an outer layer composed of PACT proteins (dPLP, PCNT), Asterless (Asl), and Plk-4 kinase. As cells transit S phase and G2 and in preparation for mitosis other proteins, such as γ-tubulin and centrosomin are recruited to the external layer. Upon mitotic entry the PCM experiences a drastic expansion in which PCNT, Cep192, and Cep215 form large extended networks. γ-tubulin, however, does not co-localize with this network suggesting additional PCM components tethering γTuRC to the PCM networks (94) (*vide supra*).

### Centriole-PCM interactions

It has become apparent that there is crosstalk between centrioles and the PCM. While centrioles appear to control protein recruitment to the PCM, the PCM may in turn regulate and contribute components to nascent centrioles. Centrosomes experience dramatic changes during the cell cycle. They double in size from S phase to mitosis, primarily through the accrual of PCM. Yet until recently little was know about the factors that control this behavior. Since the work of Bobinec et al. (102), it has been known that the PCM is under the control of centrioles. Centriole tubulins are constitutively highly glutamylated, which render them highly stable (55). Injection of anti-glutamylated α-tubulin antibodies leads to disassembly of centrioles, and remarkably, dissipation of the PCM (102). This process is reversible as cells spontaneously reassemble centrioles *de novo*, which is followed by recruitment of PCM. One centriole component in particular, Sas-4 (human CPAP), which is a component of the proximal centriole and of the complex that initiates template-dependent procentriole growth (103, 104), plays a critical role in PCM recruitment (90, 105). Defects in Sas-4 lead to abnormal centrioles and defects in PCM recruitment. Centrosomal CPAP continuously exchanges with a cytoplasmic CPAP pool (106), which is highest in G2 when recruitment of proteins to the PCM is maximal. Overexpression of CPAP results in overly long centrioles and defective PCM leading to abnormal cell division (98, 107). Expression via their own promoters of mutant forms of Sas-4 lacking the conserved PN2–3 region or defective in tubulin binding, impair (90) or promote (105), respectively, PCM recruiting, without affecting centriole duplication. These separation-of-function mutations highlight how different regions of Sas-4 critically control centriologenesis and PCM recruiting through independent domains.

## The Centrosome Cycle

**Figure 3:**
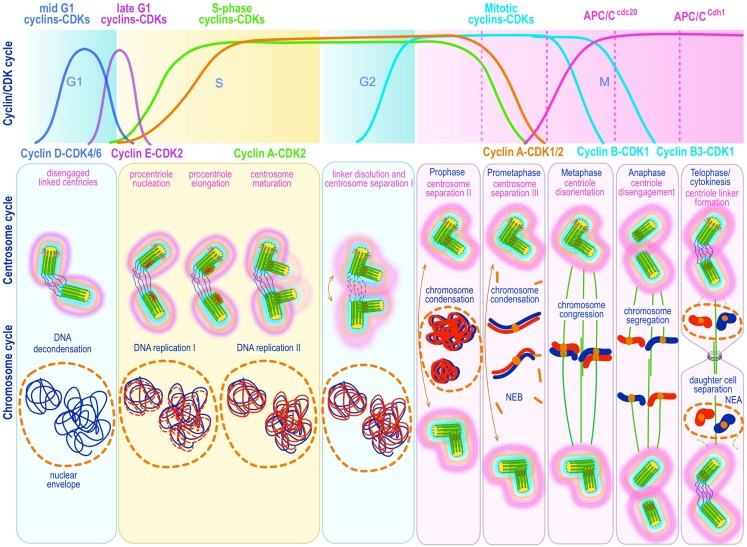
**Canonical template-dependent centrosome duplication cycle**. Normal centrosome duplication begins with centriole disengagement at the end of mitosis (anaphase-telophase). During S phase under influence of cyclin E/A-Cdk2 a procentriole is nucleated on each parental centriole, which elongate until in late S or G2 capping proteins suppress further growth. At the G2/M transition, the fibers that connect parental centrioles are dissolved while centrosomes begin to separate and mature further (acquire a full complement of PCM proteins). During prophase centrosomes continue to migrate apart until they reach opposite sides of the condensing chromosomes at metaphase and organize a bipolar spindle in preparation for chromosome segregation. The nuclear (DNA/chromosome) and CDK cycles are shown for correlation and comparison.

Centrosome replication bears remarkable similarities to DNA replication. Both are semiconservative and controlled by successive waves of cyclin E-Cdk2 and cyclin A-Cdk2 activity (19, 108–112) (Figure 3). Both occur during discrete phases of the cell cycle and both rely on licensing mechanisms for tight control and prevention of re-replication during a single cell cycle (113–117). There is general agreement that centriole disengagement – the disorientation and physical separation of centrioles at their proximal ends at the end of mitosis – is a critical early step in licensing centrosomes for replication in S phase. Lack of disengagement blocks centrosome duplication in S phase (118). Centriole disengagement, which occurs late in mitosis, is blocked by non-degradable forms of securin (119) or cyclin B1 (120), both of which block the proteolytic activity of separase, directly implicating proteolysis in this process (119, 120). Indeed, recent evidence suggests a mechanism analogous to the one operating on sister chromatid separation at anaphase, in which cohesin – the tripartite ring-like protein complex composed of Scc1, Smc1, and Smc3 – at centromeres is cleaved by separase-mediated proteolysis of Scc1. Presumably, cohesin complexes localized at the junction of engaged centrioles are proteolyzed in an analogous manner. Ectopic expression of separase, or depletion of Sgo1 – a protein that protects cohesin from separase – leads to unscheduled chromosome separation and centriole disengagement (121, 122) supporting this proposal. Nevertheless, disengagement occurs much later than sister chromatid splitting raising questions about cohesin as the exclusive target. Moreover, recent experimental work in *Drosophila* indicates that cohesin cleavage may be insufficient for centriole disengagement (123), suggesting that additional targets may exist. Two recent papers provide evidence that the relevant second substrate may be pericentrin B (PCNTB)/kendrin (124, 125). Both studies demonstrated that PCNTB is cleaved by separase at a consensus site (R2231) releasing a rapidly degraded C-terminal fragment and retaining at the centrosome the N-terminal fragment until late in mitosis. Importantly, release of the N-terminal fragment coincided with an abrupt decrease of PCNTB at the centrosome and with centriole disengagement at the end of mitosis (125). Expression of a separase cleavage-resistant form of PCNTB blocked centriole disengagement and replication, proving *in vivo* proof of its physiological relevance (124, 125). Nevertheless, the critical licensing molecular event(s) that renders a disengaged centriole competent for replication has not been elucidated.

Raising levels of cyclin A/E-Cdk2 are necessary to trigger centriole duplication (109–112), yet the initial centrosomal molecular targets of cyclin-Cdk2 that initiate/license centrosome duplication have not been defined with absolute certainty. Several candidate proteins have ben postulated to fulfill the licensing factor role. Among these, nucleophosmin (NPM1) (126–131), Mps1 (132–139), and Polo-like kinases (Plk1, 2, and 4) (118, 127, 140) figure most prominently.

The first postulated critical target of Cdk2 in triggering centrosome duplication was nucleophosmin (NPM1/B23), which is a multifunctional chaperone protein – a large fraction of which localizes to the nucleolus. NPM1 rapidly shuttles between nucleus and cytoplasm (141) and associates with the centrosome. Inactivation of NPM1 leads to unrestricted centrosome replication (142) indicating that one of its functions is to restrain centrosome duplication. Early work by Fukasawa and collaborators showed that NPM1 is one of the most conspicuous targets of Cdk2 in unduplicated centrosomes and that phosphorylation of NPM1 on T199 by Cdk2-cyclin E leads to its dissociation from centrosomes (129, 130). Careful immunofluorescence with an antibody specific to centrosomal NPM1 showed it to localize between the centrioles in the unduplicated centrosome, to dissociate from centrosomes upon its phosphorylation, and to re-associates with the centrosome during mitosis (129, 143, 144). Based on these observations a centrosome duplication licensing role for NPM1 has been proposed (129). Phosphorylation of T199 and S4, have both been postulated as critical events for NPM1 downstream functions. Both Cdk2 and Plk1 phosphorylate T199 creating high-affinity docking site for the kinase ROCK-II, which becomes “hyperactivated” upon binding NPM1 (145). Activated ROCK-II, reportedly bypasses the Cdk2 requirement for centrosome replication (145). But NPM1 is also phosphorylated at S4 by Plk1 during mitosis (146) and by Plk-2 near the G1/S transition (147, 148). Plk1 S4 phosphorylation in coordination with separase contributes to centriole disengagement (118), while Plk-2 S4 phosphorylation has been proposed to be the trigger for centrosome replication (127), based on the fact that NPM1 S4A – a non-phosphorylatable form of NPM1 – blocks centrosome duplication, while phosphomimetic mutants have the opposite effect, i.e., centrosome overreplication (127). Clearly, further experimentation will be required to precisely map the role of NPM1 in centrosome duplication.

Mps1 (Esk) (132, 133, 136, 149) overexpression in S-arrested mouse cells leads to centrosome overduplication, while a kinase-dead form blocks centrosome duplication (133). Similarly, overexpression of a dominant negative form of Msp1 in human cells blocks centrosome duplication, while active Mps1 overexpression accelerates centrosome duplication (132). Notably, siRNA Mps1 knock-down blocks centrosome duplication and in addition cause pleiotropic defects resulting in severe mitotic abnormalities, attesting to Mps1 regulatory function in many mitotic processes, in particular on the spindle assembly checkpoint (132). Mps1 normally is under negative regulation by proteosomal destruction. Preventing the degradation of Mps1 by transient exposure to proteasome inhibition is sufficient to cause centrosome reduplication in human cells (136). Phosphorylation of Mps1 T468 appears to be the critical target of Cdk2 leading to proteasome resistance and Mps1 accumulation, since phosphomimetic mutations of T468 prevent Mps1 degradation and lead to Cdk2 independent centrosome duplication (136). This process seems to be more nuanced in human cells. Early studies showed that hMps1 was only detectable at kinetochores but not at centrosomes, and that neither overexpression of hMps1, kinase-dead hMps1, or siRNA knock-down of endogenous hMps1 in human cells revealed a centrosome phenotype, casting doubts into an hMps1 role in centrosome duplication (138). Nevertheless, GFP-hMps1^T468A^, a non-phosphorylatable form of hMps1, accumulates in the cytoplasm but is continuously removed from centrosomes in a proteasome-dependent manner (136), suggesting that it is the centrosomal pool of hMps1 the one relevant for the hMps1 centrosome phenotype. In contrast hMps1^T468D^ and hMps1^T468E^, mutants that mimic T468 phosphorylation, and hMps1^delta12/13^ that lacks an Mps1 degradation signal (MDS), readily cause centrosome reduplication, even in the absence of cyclin A-Cdk activity. Accordingly, failure of wild type hMps1 overexpression to cause centrosome re-duplication appears to be the consequence of its efficient proteasome-dependent removal from the centrosome (137). The exquisite control of centrosomal hMps1 appears to depend largely on its MDS signal. Yet the MDS signal bears no resemblance to known targeting motifs for SCF or APC/C type E3-ubiquiting ligases. Emerging evidence suggest that hMps1 degradation may be controlled by ornithine decarboxylase antizyme (OAZ) (135). OAZ target substrates for ubiquitin-independent proteasome-mediated degradation (150). It has been demonstrated that OAZ localizes to the centrosome and that its activity suppresses centrosome re-duplication, while reducing OAZ at the centrosome leads to centrosome re-duplication (151). Indeed OAZ binds hMpsi1 through its MDS motif and leads to its degradation through proteasome-dependent proteolysis. Yet one additional mechanisms control the level of hMps1. A single N-terminal D-box makes hMps1 a target for APC/C-dependent degradation during mitosis, an activity that is controlled by Cdc20 and Cdh1 (152). It is possible that this second hMps1 degradation mechanism is used at the end of mitosis to rapidly reset hMps1 to low levels before reaching the G1/S transition, where the finer regulation effected by OAZ takes over (137).

Polo-like kinases have also been considered key factors in centrosome replication, possibly playing a licensing role as well. It has long being known that in addition to separase, Plk1 activity is required for centriole disengagement at the end of mitosis (118). Moreover, the recently described phosphorylation of NPM1 S4 has been proposed as a centriole duplication trigger (127). Additionally, new procentrioles require a Plk1 dependent modification, which can only occur through mitotic passage, when Plk1 activity is high. This prevents the growth of “granddaughter” centrioles, i.e., procentrioles growing from daughter centriole walls within the same cell cycle (153, 154). Plk-4 has also received considerable attention as a potential centrosome replication licensing factor (155–157) [reviewed in (140, 158)]. Plk-4 activity peaks only transiently during mitosis and is kept at very low levels during interphase (156, 159) by autoregulatory self-destruction. Plk-4 homdimerizes and autophosphorylates in trans, which triggers rapid SCF^limb^ E3 ubiquitination and proteasome directed proteolysis (158–162) enforcing low activity levels through most of the cell cycle. Plk-4 peak activity in mitosis is due to its interaction with Twins – the regulatory subunit of protein phosphatase 2A (PP2A), which in complex with PP2A dephosphorylates Plk-4, briefly spearing it from proteolysis (156). Plk-4/Sak activity is required in the earliest steps of procentriole formation (97, 155, 157, 163), and is recruited to the centrosome together with Cep152 (homolog of *D. melanogaster* Asterless) through interactions with Cep192 (164) (homolog of *C. elegans* Spd-2). The complex localizes to the outer surface of the proximal end of the mother centriole, precisely the site from which the procentriole will sprout (97). Thus, Plk-4 satisfies most criteria for a licensing factor for centriole duplication (116): Plk-4 levels peak only once during the cell cycle in mitosis; Plk-4 is kept low during all other stages of the cell cycle by autoregulatory phosphorylation-triggered proteolysis; increasing levels of Plk-4 experimentally leads to centriole reduplication; and, decreasing Plk-4 or suppressing its activity prevents centriole duplication (165).

Centrosome replication begins at the G1/S transition with nucleation of a procentriole at the base of the parental centrioles (108, 166–169) [reviewed in Ref. (170–172)]. Of these Plk-4 (97), Cep152 (97), and SAS-6 (172) initiates the process, with Plk-4 participating as the dominant kinase and regulator of the early steps of centriole duplication. By superresolution microscopy, Plk-4 initially localizes to a single spot within the toroid defined by N-terminal Cep152 at the proximal end of the mother centriole, but outside the one defined by C-terminal Cep152, which is consistent with the known interaction between Plk-4 and the N-terminal Cep152 (173–175). Interestingly, Plk-4 is detectable in G1 cells at the spot on the mother centriole from which later Sas-6 will initiate cartwheel assembly formation. This observation suggest that Plk-4 determines the site of initiation of procentriole formation.

SAS-4 may be important in recruiting Cep152, a PCM protein that participates in nucleation of procentrioles (164, 176). The interaction of Cep152 with Sas-4/CPAP may provide the initial scaffold for procentriole formation (147, 174). Phosphorylation of the F-box SCF^Fbxw5^ E3-ubiquitin ligase by Plk-4 appears to stabilize SAS-6, a natural substrate of SCF^Fbxw5^, initiating procentriole growth (177) (Figure 3). The first structure to appear at the site of procentriole formation, even before microtubules nucleation is apparent, is the cartwheel assembly. Mutations in cartwheel constituents such as SAS-6 – the central component of the cartwheel driving the establishment and maintenance of the ninefold symmetry of the centriole (178, 179) – lead to absence or severely defective centrioles. In human cells the first protein known to localize to the procentriole is indeed HsSAS-6 (180). SAS-6 molecules have conserved amino-terminal domains, followed by coil–coil domains capped by poorly conserved carboxy-terminal domains. SAS-6 homodimerizes in parallel via the coil-coil domains resulting in a rod-like structure in which the conserved globular amino-terminal domains are located next to each other at one end of the rod-like dimer. Interactions of one of these domains with a similar domain in a second homodimer leads to the progressive assembly of the cartwheel, with the globular amino-terminal domains constituting the central hub, and the coil–coil homodimers the centrifugally radiating spokes of the cartwheel assembly (181, 182) (Figure 2). The self-assembly of the cartwheel starting from homodimers of SAS-6 is a remarkable effective biological organizing principle that satisfies the need of ninefold symmetry in the simplest possible manner (182–184). SAS-6 is known to interact with SAS-5/Ana2/STIL, but the nature and consequences of the interaction are not well defined. It has ben proposed that SAS-5/Ana2/STIL interacts with SAS-6 through a STAN motif, stabilizing the procentriole cartwheel structure (185–188). Overexpressed SAS-6 and Ana2 in flies co-assemble into long cartwheel structures closely resembling the natural structure (188). Two safety mechanisms are put in place to avoid overreplicating the cartwheel structure. During S and G2 phases the SCF^Fbxw5^ E3-ubiquitin ligase is inhibited by Plk-4 mediated phosphorylation. As the cell cycle progresses Plk-4 autophosphorylates, triggering its own proteolysis, and relieving the inhibition of SCF^Fbxw5^, which then ubiquitinylates Sas-6 triggering its degradation (177). A second safety check is active in mitosis where Sas-6 is ubiquitinylated by APCC^cdh1^, which targets it for proteolysis ensuring low levels of Sas-6 throughout mitosis (180). This tandem safety mechanism prevents re-initiation of centriole duplication once a daughter centriole has emerged.

Superresolution microscopy tracing the localization in S/G2 cells of Sas-6, Cep135 and STIL, three proteins involved in template-dependent procentriole formation, shows that Sas-6 and STIL co-localize precisely (189–191), whereas Cep135 localizes away from Sas-6/STIL in a position similar to C-Nap1, a marker of the proximal end of mother centriole (170). In late G2 and M phase cells, however, Cep135 staining could be seen to extend into the area occupied by Sas-6/STIL indicating that Cep135 progressively associates with the proximal end of growing daughter centrioles. Both Sas-6 and STIL are degraded upon exit from mitosis (180, 189–191) and are no longer detectable in the centrioles from early G1 cells. Unresolved still are the mechanism that propagate the cartwheel at the proximal end of the centriole, which could occur by deposition of cartwheel structures in layers or by a helical mechanism resembling the bristles of a bottle brush, the mechanisms that limits the growth of the cartwheel, and the interacting proteins that regulate the self-assembly and precisely control the angle of interacting SAS-6 homodimers to ensure ninefold symmetry. The initial structure is stabilized further by SAS-4/CPAP, which also plays an important role in recruiting microtubules to the perimeter of the growing cartwheel structure (90, 103, 104, 107).

Microtubules are next added to the cartwheel in an orderly fashion with nucleation of the A microtubule initiated at a cone-like structure attached to the distal carboxy-terminal end of the radiating SAS-6 spokes and proceeding unidirectionally to the distal end of the nascent centriole (192). The cone-like structure may contain γ-tubulin and its interacting partner NEDD1, because depletion of these components prevents centriole growth (72). The B and C microtubules apparently require δ-tubulin and ϵ-tubulin (193, 194) and appear to polymerize bi-directionally. Additional proteins participate in the regulation and addition of microtubules to the emerging centriole. Amongst these CPAP and STIL form a complex with SAS-6 and are likely to contribute to microtubule addition (190). CPAP localization to the procentriole is dependent on phosphorylation by Plk-2 (195). Procentrioles, securely attached to their mothers, grow by elongation through G1, S, and G2 phases (Figure 3). Centriole elongation and final length are specifically controlled, and are characteristic of a species and cell type. There appears to be distinct proximal and distal elongation steps that are independently regulated. Distal growth may be regulated at least partially by the centrin-binding protein hPOC5 (proteome of centriole 5) since its depletion prevent distal but not proximal elongation (196). Conversely, CPAP, CP110, and POC1 may control proximal elongation. Overexpression of CPAP (98, 107, 197) or POC1 (198), or depletion of CP110 (98) leads to unusually long daughter centrioles. Conversely, depletion of POC1 (198) or overexpression of CP110, prevent procentriole elongation and leads to centriole overreplication (98). siRNA depletion of Cyclin F, which interacts with CP110, leads to CP110 overexpression and centrosome overreplication (46). Cyclin F is part of the ubiquitin ligase SCF^cyclinF^, which targets substrates for proteasome degradation. A recently described deubiquitinating enzyme named USP33 (199) specifically interacts with CP110 bound to centrioles in late S and G2/M cells, protecting it from SCF^cyclinF^. Depletion of USP33 leads to decreased levels of CP110 (199) and centriole elongation. Remarkably, no other centrosome SCF^cyclinF^ substrate is also a substrate of USP33, attesting to the importance of fine-tuning the levels of CP110 in centriole replication (199). The process is completed with the distal binding of the capping proteins CP110 and Cep97, which prevent further growth of the procentriole, determining its final length (97, 98, 200, 201) (Figure 2).

Initially devoid of PCM, the daughter centriole rapidly matures, i.e., acquires PCM and the increased ability to nucleate microtubules, by the end of G2 and in mitosis, through the concerted actions of the kinases Aurora-A and Plk1 which reach high levels at the centrosome at the end of G2 (202–204). In a manner highly reminiscent of its priming function in DNA replication (205, 206), Plk1 induced centrosome maturation is essential for priming procentriole nucleation in the next cell cycle (118, 153, 154).

At the G2/M transition in a process termed centrosome disjunction, centrosomes begin to separate by dissolving the linker protein fibers connecting the proximal ends of the two parental centrioles, which were established previously around the time of centriole disengagement in late M or early G1 (207) (Figure 3). Dissolution of the linker fibers is dependent on Nek2 activity. Nek2 is a NIMA-related kinase that accumulates at the centrosome through S and G2 phases. At the G2/M transition it triggers dissolution of the intercentriolar linker. The importance of Nek2 in this process is illustrated by changing Nek2 levels at the centrosome. Overexpression of Nek2 leads to premature separation of centrosomes (170) while knock down with siRNA inhibits centrosome separation (208). Two main protein components of the linker fibers, C-Nap1 and rootletin, are phosphorylated by Nek2A (170, 209), promoting their migration onto the fibers. While rootletin appears to distribute uniformly throughout the fibers the Nek2 and C-Nap1 localize mostly to the proximal ends of the mother centriole (170) suggesting that they are docking sites for rootletin (209) (Figures 2 and 3). The levels of rootletin itself may control the length of the fibers. Overexpressing this protein lengthens the fibers (210, 211), while depleting it results in premature centrosome separation (212). Two additional putative linker proteins, Cep68 and Cep215 (CDK5RAP2), have been described, the former being a bona fide substrate of Nek2A and the latter possibly of Plk1 (213). By high-resolution fluorescence/deconvolution microscopy and immune-EM, Cep68 is seen to form fibers that attach to the proximal end of mother centrioles, while Cep215 instead, tightly surrounds the mother centriole (213). siRNA knockdown of either induces premature centrosome separation (213) implicating them in centrosome cohesion. However, overexpression of Cep68 does not induce fiber formation by itself but is readily recruited to rootletin fibers (213) indicating that Cep68 cooperates with rootletin and C-Nap1 in centrosome cohesion. Cep215 neither distributes to the linker, nor interacts with rootletin or C-Nap1, indicating that it does not represent a *bona fide* linker protein. Instead, its centrosome cohesion promoting function may be related to its interaction with pericentrin (213). Since Nek2 activation promotes centrosome separation by evicting linker components, it needs to be tightly regulated to prevent premature separation of centrosomes. Nek2 becomes activated by homodimerization through its coil–coil motifs, which facilitates autophosphorylation of the catalytic domain (214, 215). Further insight into Nek2 regulation has come from the discovery of KVHF motifs in its non-catalytical C-terminal domain. KVHF motifs are consensus sequences for the binding of Protein phosphatase 1 (PP1). PP1a dephosphorylates the catalytic domain of Nek2 and inactivates it. Notably PP1 itself can be inactivated by Nek2 mediated phosphorylation (214, 215). This on-off bi-stable switch is characteristic of many of the regulators that control abrupt mitotic transitions. The balance of this metastable switch appears to be tipped by the Inhibitor-2 protein (216), which binds and inhibits PP1 leading to runaway auto activation of Nek2.

Upstream regulators of the cell cycle directly control timely activation of Nek2. The main function of Aurora-A in centrosome separation is activation of Plk1 kinase by phosphorylating T120 in the T loop (217–219). Activated Plk1 phosphorylates and binds Mst2 kinase, which can now bind Nek2A and PP1γ (215, 220). Unlike PP1α, PP1γ antagonizes Nek2, not by direct dephosphorylation, but by dephosphorylating C-Nap1, its binding partner. The level of Plk1 phosphorylated Mst2 ultimately determines the dissociation of the Mst2-Nek2A-PPIγ complex (221) with increasing phosphorylated Mst2 leading to a reduction in PP1γ on the complex. Thus, phosphorylation of Mst2 by Plk1 leads to a reduction in the levels of PP1γ in the Mst2-Nek2A-PP1γ complex resulting in increased Nek2 dependent phosphorylation of C-Nap1 and dissolution of centrosome linker fibers (221). This Hippo dependent increase in Nek2 activity is counteracted by pericentrin and HEF1, the latter a focal adhesion scaffold protein. While pericentrin is an inhibitor of Nek2 kinase activity, HEF1 inhibits accumulation of Nek2 at the centrosome (222). siRNA knockdown of pericentrin causes premature separation of centrosomes in interphase. It has been proposed that pericentrin changes the structural conformation of Nek2 catalytic domain into an inhibitory conformation (223). Pericentrin and Cep125 localization to the centrosome also depends on Plk1 activity suggesting an additional level of regulation, either indirectly through Nek2A or by direct phosphorylation of some proteins involved in centrosome cohesion (213, 224).

After dissolution of the centriole linker, motor proteins bind anti-parallel astral microtubules and exert their sliding forces by walking toward MT plus ends (Figure 3). The kinesin Eg5 is the principal force generator for centrosome separation at this stage (225–229). Eg5 is a homotetrameric plus-end directed motor belonging to the kinesin-5 subfamily (230, 231). Knockdown of Eg5, by siRNA or chemical inhibition with monastrol, arrest cells in prometaphase with monopolar spindles (232, 233). Mainly cytosolic during interphase, Eg5 rapidly accumulates at spindle poles in prophase (234). There is evidence that Plk1 phosphorylation of Eg5 targets it to the spindle poles (235–237). Inhibition of Plk1 prevents accumulation of Eg5 at the centrosome, but does not change the overall level of cytoplasmic Plk1 (236). Cdk1 phosphorylates Eg5 at T927 (238). Plk1 can substitute for Cdk1. However, Plk1 phosphorylated Eg5 triggers slow and erratic centrosome separation, while Cdk1 triggers fast movement. The difference in centrosome behavior under these conditions has been attributed to differential modulation of microtubule dynamics by Cdk1 and Plk1 (237). Plk1 induced accumulation of Eg5 at the centrosome is microtubule dependent and is abolished in the presence of nocodazole, a standard MT depolymerizing agent (236). The effect of Plk1 on Eg5 may indeed be associated with the ability of Plk1 to increase the capacity of centrosomes to nucleate MT (204). Other members of the NIMA kinase family also participate in Eg5 regulation. There is experimental evidence that suggest phosphorylation of S1033 in Eg5 by Nek6 is the critical event that targets Eg5 to the centrosome after Plk1 activation (235). Tellingly, mutations in Eg5 that prevent S1033 phosphorylation abolish Eg5 localization to the centrosome (235). Moreover, differential regulation of Eg5 targeting to the centrosome, before and after NEB, appears to exist (239). Indeed, recent experimental evidence seems to validate this proposal (240). It has long been known that there are two pathways to mitotic spindle assembly. A prophase pathway occurring entirely before NEB, and a back-up pathway that occur in prometaphase, after NEB (241–246), reviewed in Ref. (225, 228). Importantly, the back-up prometaphase pathway is mechanistically more complicated and more likely to lead to chromosome segregation errors than the prophase pathway (247). Moreover, the two pathways are temporally, spatially and genetically distinct (240). These differences may be important in that the probability of a mitosis generating abnormally attached chromosomes depends on avoiding kinetochore MT from the two spindle poles contacting the same kinetochore. In the prometaphase pathway centrosomes are incompletely separated when astral microtubules can first contact kinetochores after NEB, increasing the chances of chromosomes with merothelic attachments to the spindle poles. This has indeed been demonstrated in PtK1 cells (247). In conclusion, it appears that whether a cell uses the prophase or the prometaphase centrosome separation and spindle assembly pathways, may have important consequences in the form of increased CIN in the latter. Incompletely separated centrosomes represent a mitotic liability that may translate into CIN. Nevertheless, the potential contribution of abnormal centrosome separation to CIN in cancer has not been directly studied.

A recent detailed *in vivo* imaging study in Eg5-mEGFP/mCherry-α-tubulin in HeLa cells in which the kinetics of centrosome separation could be precisely followed and cells indexed as either prophase or prometaphase centrosome separation pathway users, revealed interesting results (240). Cells using the “back-up” prometaphase pathway had longer lags, and lower velocity and a shorter centrosome translocation times. Of 1,388 mitotic events studied, half used the prophase centrosome separation pathway, and the other half used the prometaphase pathway (240). Whereas 0.7% of cell utilizing the prophase pathway had lagging chromosomes at metaphase (a surrogate of CIN), as many a 2.3% cells using the prometaphase pathway exhibited the same phenotype (240). It would be of great interest to repeat this experiments in immortalized non-transformed cells, since HeLa were derived from a uterine cervix carcinoma and exhibits measurable CIN under standard growth conditions [see Ref. (248) and references therein] and also to study this phenomenon in additional cancer cells. During metaphase centrosomes complete maturation achieving maximal microtubule-organizing capacity, and together with kinetochores, assemble the mitotic spindle. Late in mitosis (anaphase, telophase) daughter centrioles disengage from their mothers reinitiating he centrosome replication once again.

## Abnormal Centrosomes in Cancer

Theodore Boveri, who co-discovered the centrosome, was the first to propose that centrosomes may induce CIN, which could lead to cancer (6). Boveri’s cancer development theory was singularly influenced by observations made by his colleague Leo Hansemann. Hansemann had observed abnormal mitoses in cancer tissue and had compiled beautiful renderings of their salient features (4, 5) (*vide supra*), which to Boveri’s trained embryologist eye, immediately suggested the work of abnormal centrosomes. Boveri theorized that a peculiar “combination of genetic determinants” may give rise to cancer and the transmission of that peculiar combination of genetic determinants (i.e., genes) may occasionally arise in daughter cells of abnormal multipolar mitoses. Boveri reasoned that the degree of centrosome abnormalities he inferred from Hansemann’s drawings, were probably detrimental as a whole, as he had directly observed in doubly fertilized sea-urchin zygotes (249). He theorized that only cells exhibiting lesser degrees of centrosome malfunction could propagate the cancer phenotype, which he ascribed directly to the genetic material (6).

Abnormal centrosomes in cancer were initially detected in some of the most common human cancers including breast, colon, lung, and brain cancer (250, 251). These findings were quickly confirmed and expanded by others (252–263). A century after Boveri and Hansemann original observations, we can affirm that centrosome defects are indeed pervasive in cancer. Excepting leukemias and some low-grade lymphomas, most carcinomas and sarcomas, and a subset of high-grade lymphoma [for a survey of clinical cancer types, see Ref. (264)] exhibit abnormal centrosomes. Furthermore, it is now well established that centrosome abnormalities in cancer correlate closely with and are an important cause of CIN (251) [for a review, see Ref. (265–268)], and that centrosome abnormalities and CIN frequently co-occur in carcinoma in situ (269, 270). These observations place centrosome abnormalities at the earliest stages of cancer development and argue against a purely secondary role or late effect. Nevertheless, despite their common occurrence, and perhaps due to the heterogeneity of centrosome abnormalities in cancer, it has been difficult to determine whether centrosomes abnormalities are caused by primary intrinsic centrosome defects, or are the consequence of dysfunction of other cellular processes that lead to the accumulation of normally replicated centrosomes, such as for instance in cases of cell division failure (271, 272). In consequence a considerable volume of ongoing research is being devoted to elucidating in detail the molecular pathways involved in centrosome dysfunction in cancer, their impact on the cancer genome, and the prospects of utilizing centrosome defects as biomarkers (205, 273, 274) and targets for cancer specific therapy (275–279).

## The Spectrum of Centrosome Abnormalities in Cancer

Centrosome phenotypes in cancer are heterogeneous with both numerical and structural abnormalities documented. Nevertheless, attempts at classification of centrosome abnormalities in cancer have met with limited success, primarily because of the difficulty inherent in carrying out comprehensive surveys at the ultrastructural level using EM, which until recently has been the only technology capable of visualizing centrioles and PCM with sufficient detail (280–283). The emergence of superresolution microscopy promises to drastically change the *status quo*. Structured illumination microscopy is already providing images of whole centrosomes with unprecedented resolution and is poised to contribute greatly to our understanding of centrosome phenotypes in cancer in the immediate future (85) (*vide supra*).

**Figure 4:**
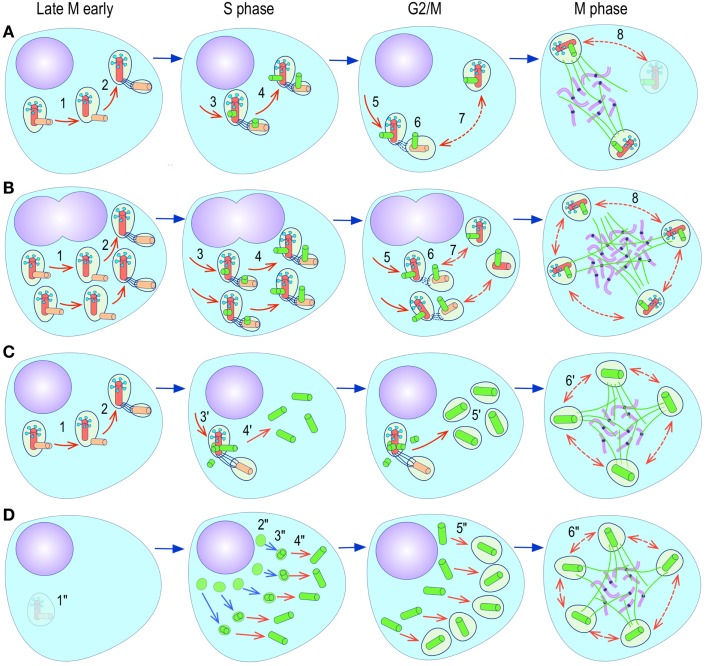
**Pathways to supernumerary centrosomes in cancer**. Canonical template-dependent centrosome replication pathway **(A)**. Normal centrosome duplication proceeds sequentially in the following steps: centriole disengagement (1), linker fiber development (2), procentriole nucleation (3), centriole elongation (4), linker dissolution (5), centrosome maturation (6) and separation, before (7) and after (8) NEB. There are at least three pathways to supernumerary centrosomes in cancer **(B–D)**. Centrosome accumulation pathway due to polyploidization events **(B)**. Events such as cytokinesis failure, mitotic slippage (mitotic failure before cytokinesis), etc., with or without normal DNA replication, result in accumulation of normally replicated centrosomes, which execute all stages of replication as in the canonical template-dependent pathway A. Centrosome over-replication pathway **(C)**. Some cancer cells, particularly if arrested in S or G2 phases, such as during DNA replication stress induced by hypoxia, chemotherapy or radiation therapy, undergo multiple rounds of templated centriole duplication (3′), which subsequently elongate (4′) and mature (5′) leading to functional centrosomes capable of enacting multipolar mitoses (6′). *De novo* centriole formation pathway **(D)**. Under similar conditions certain cancer cells, even when containing resident centrosome, build new centrioles *de novo*, via centriole satellites (2′′). Once synthesized such centrioles (3′′) can elongate (4′′), mature by acquiring normal mitotic PCM (5′′), and become competent at mitosis (6′′) usually enacting multipolar spindles. In subsequent cell divisions, *de novo* centrosomes are thought to replicate via the canonical template-dependent pathway.

The most evident and widely documented centrosome cancer phenotype is supernumerary centrosomes (250, 251) (Figure 4). In principle, supernumerary centrosomes may result from at least three separate mechanisms: template-mediated over-replication of pre-existing centrosomes within one cell cycle (hereby termed the *over-replication* pathway), a phenotype that has been variably referred to as centrosome amplification (254, 263, 284–286) or hyperamplification (252, 255, 287, 288), *de novo* formation during interphase (*de novo* pathway) (289, 290) (Figure 4) or from accumulation of normally (or abnormally) replicated centrosomes due to failed cell division after replication of centrosomes and chromosomes has occurred (*accumulation pathway*) (291, 292) (Figure 4) [reviewed in Ref. (293, 294)]. While in the latter the normal numerical relationship of one centrosome per diploid chromosome set in G1 phase is maintained, in the former two pathways it is halved or worse. This difference profoundly affects the chances of daughter cell survival in cells carrying multipolar mitoses to completion, since daughter cell viability is predicated on receiving at least a full haploid set of chromosomes. This in fact is one of the seminal experimental observations made by Boveri in dispermic see urchin eggs, which allowed him to infer that chromosomes are not interchangeable and therefore must carry different genetic determinants (249).

It is likely that only one of these three centrosome amplification pathways operates in an individual tumor. Nevertheless, there is precedent for pathway cooperation. Using a marker for the mother centriole, Duensing et al. were able to determine that the papillomavirus oncoprotein HPV16-E7 leads to over-replication of centrosomes in G2, while HPV16-E6 leads to both increased centrosomes and increased ploidy (295), by a mechanism that apparently involves cytokinesis failure.

## *In vivo* Centrosome Biogenesis in Cancer and Non-Cancer Cells

In a series of seminal observations that rekindled interest in centrosome biology in cancer, Fukasawa et al were the first to note that p53 null mouse embryo fibroblasts (MEFs) in culture frequently acquire supernumerary centrosomes (285). The supernumerary centrosome phenotype of p53 null MEFs was exacerbated by forced overexpressing of Cyclin E, a cyclin known to promote centrosome replication (288). Furthermore in an assay that measures the entrainment of centrosome replication to the DNA replication cycle by blocking cells at the G1-S boundary with a DNA replication inhibitor, the authors could demonstrate that in p53 wild type cells, only one round of centrosome replication occurred, while in p53 null MEFs, and to a lesser extent in Waf1 null MEFs, centrosome continued to replicate several times (296) (centrosome over-replication pathway). Furthermore, restoring p53 to p53^−/−^ MEFs restored normal centrosome replication control. These experiments clearly demonstrated that p53 played a major role in centrosome homeostasis, and that prevention of centrosome over replication was clearly dependent on an intact p53-Waf1 axis (296). These experiments however did not offer a glimpse as to the actual pathway leading to supernumerary centrosomes in continuously proliferating p53 deficient MEFs. More direct evidence for a plausible mechanisms of supernumerary centrosomes in cycling p53 null cells was obtained by overexpressing a number of mitotic kinases (Aurora-A, polo-like kinase 1 (PLK1), Aurora-B, or Aurora-C kinases) in a p53 wild or null context. High level of Aurora-A or Plk1 lead to supernumerary centrosomes via defects in cell division resulting in tetraploidization and centrosome accumulation (292, 297) (Figure 4). These results suggested that the supernumerary centrosome phenotype of p53 null cells documented by Fukasawa et al (285) in normally dividing MEFs was most likely due to polyploidization and accumulation of centrosomes rather than over replication (292). However, it is important to make clear from the outset that cell division failure, as a cause of centrosome amplification is only a viable tumorigenic mechanism insofar as it may be intermittent and stochastic, occurring only in a minority of cell divisions. Sustained division failure leads to exponential chromosome and centrosome accumulation with giant cell formation, which is detrimental to tumor growth. Accumulation of centrosomes and subsequent multipolar spindles is an attractive explanation for supernumerary centrosomes in p53 null MEFs, because it is consistent with one of the known checkpoint functions of p53, which is to impose a G1 arrest in response to tetraploidization (298–300). Absence of this p53 checkpoint explained why p53 null MEFs with extra centrosomes continued to divide and enact multipolar spindles. Moreover, these observations fit in well with the transient tetraploid state know to occur in many cancers before aneuploidy ensues (301).

Although tetraploidy and centrosome accumulation may be a prevalent pathway to supernumerary centrosomes in cancer, there is precedent for centrosome over-replication in especial situations. For instance, overexpression of PLK-4 in Hela cells arrested in S phase with aphidicolin leads to over replication of centrosomes, with multiple procentrioles attached to the mother centriole (97, 157). A similar phenotype can be induced by overexpression of other core components of the template-mediated centriole replication pathway, such as HsSAS-6 (106, 189–191, 302). However, even non-cancer cells may respond differentially to perturbations in the centrosome replication pathway depending on tissue of origin or differentiation. For instance, overexpression of DSas-6 in *Drosophila* leads, within on cell cycle, to a fraction of centrosome undergoing template-dependent over replication in syncytial embryo and somatic brain cells, *de novo* assembly of multiple centrosomes in eggs, and no abnormality in spermatocytes (303). These studies reinforce the concept that normal cells use the *de novo* assembly pathway only in the absence of resident centrosomes such as it exist in eggs. The need for tight control of the core proteins involved in centriole duplication and their regulatory kinases is again exemplified by PLK-4. While high levels of PLK-4 leads to multiple procentrioles (97, 157), insufficient levels of PLK-4 is associated with abnormal centrosomes with reduced microtubule nucleation capacity, abnormal spindles, and CIN (304, 305).

Most cancer cells, but not normal untransformed cells, experience centrosome over replication upon prolonged interphase arrest. This phenotype is very relevant in oncology since cancer chemotherapy, which often includes DNA replication inhibitors, could potentially cause centrosome amplification, furthering genomic instability. Competence for centrosome over replication in cancer cells arrested in S phase appears to be conferred, in addition to Plk-4 which triggers procentriole formation, by procentriole maturation normally induced by active Plk1^pT210^ at the S to G2 transition (153). Prolonging interphase, particularly G2 phase leads to procentriole maturation and disengagement allowing for a second procentriole to form, which in a background of persistently high Plk1^pT210^, matures and disengage perpetuating the over-replication cycle (153). Nevertheless, Plk1 is dispensable for centriole formation and appears to participate only in centrosome maturation/disengagement, thus coordinating the cell cycle with procentriole maturation (153). Cells arrested in S-phase by depletion of a mitotic inhibitor (early mitotic inhibitor 1, Emi1) assembled procentrioles, which do not grow further unless Plk1^pT210^ is available. Plk1^pT210^ peaks at the beginning of G2 fulfilling its role as centrosome maturation factor. Plk1^pT210^ does not localize to the centrosome until late S early G2 since it is not present in HU S-phase arrested cells (217) but is found at the centrosome in G2 arrested cells, all of which is consistent with Plk1^pT210^ being critical in the centrosome cycle in G2 (153).

*De novo* centrosome formation (Figure 4) is known to normally occur only in lower eukaryotes (306), in eggs (307), and in parthenogenetic embryos (308). Although it has never been documented in normal vertebrate cells, other than in Chinese hamster ovary (CHO) cells, it may occur in cancer cells under special circumstances. In CHO cells arrested in S phase by hydroxyurea, destruction of centrosomes by laser microsurgery leads to the appearance of new PCM clouds 5–8 h after ablation (289). The clouds of PCM contain γ-tubulin, pericentrin (components of the PCM), and ninein (centriole). By 24 h clouds appeared more compact and exhibited a central denser area where a centriole was located. As many as 14 centrosomes per cell developed in cells arrested in S-phase for 24 h. Of note, PCM accumulation and accretion into denser clouds was not dependent on microtubules. Only the development of centrioles was, since nocodazole pre-treatment abolished centriole formation but not PCM accumulation (289). Most centrioles had normal EM structure but some were aberrant partially open centriolar cylinders, distorted/bent walls, and different cylinder lengths. These structures are very similar to those observed during centrosome reassembly after loading cytoplasm with anti-polyglutamylated tubulin antibody a maneuver that leads to centrosome dispersal (102). In general all newly formed centrosome clustered together near the nuclear envelope and only rarely were they dispersed or away from the nuclear envelope. Interestingly, ninein distribution volume within the PCM clouds was restricted to a single dot located next to the single area of greatest γ-tubulin density, a location that suggest its normal presence in the distal end of the centriole. Nevertheless, there were no obvious appendages on the neocentrioles when examined by electron microscopy. These structures are able to nucleate microtubules and are thus functional. When cells with neocentrosomes were allowed to reenter mitosis by HU washout and exposure to caffeine, which induced rapid entry into mitosis, the majority of the cells assembled multipolar spindles, attempted multifurrow cytokinesis, which failed to complete, resulting in single daughter cells. Only two cells with multipolar spindles resulted in two (one) or three (one) daughter cells. Importantly, *de novo* centrosome formation in non-transformed mammalian cells does not occur in the presence of a single pre-existing normal centrosome. Taken all these data together it is reasonable to conclude that the template-dependent centriole duplication pathway is dominant, and the *de novo* centrosome formation pathway is only enacted if no centriole template is available to the cell. For instance, CHO cells with intact centrosome subjected to the identical S-phase block replicate centrosomes every 20 h and only using the template-dependent mechanism (309). One can conclude that *de novo* centrosome formation is a default back-up mechanism for cells that no longer have functional centrosomes. How the presence of centrioles suppresses the *de novo* pathway is currently unknown.

The significance of the *de novo* centrosome assembly pathway in cancer has been difficult to ascertain. For once, removal of the centrosome from normal, untransformed vertebrate cells leads to cell cycle arrest in G1 without centrosome neoformation (48, 310). Selective ablation of a single centrosome suppresses *de novo* centrosome formation in the daughter cell receiving the non-ablated centrosome indicating that an active pathway exist to suppress *de novo* centrosome formation. However, transformed cells such as HeLa cells lack such a checkpoint. Removal of resident centrosomes by laser ablation or micromechanical manipulation in HeLa cells does not result in G1 arrest, instead cells progress through mitosis and into S phase assembling centrosomes *de novo* (290). *De novo* centrosome assembly begins at the G1/S transition as faint centrin dots, which become recognizable centrosomes before mitosis. Remarkably, such centrosomes are immature, i.e., do not nucleate full arrays of microtubules, until the next cell cycle suggesting that cell cycle progression is necessary for completion of centrosome maturation. After the second mitosis, neocentrosomes coalesced into a single focus, evinced prominent PCM, and were associated with the main microtubule array focus indicating that they are by now fully competent centrosomes (290). Interestingly, *de novo* assembly of centrosome does not occur in cells arrested in G1 phase after centrosome ablation, but it does in cells arrested in S phase, indicating that the *de novo* pathway is only turned on in S phase, the cell cycle phase where normal, i.e., template-dependent centriole duplication occurs. Whether a mother or daughter centriole can suppress the *de novo* pathway was tested by specifically ablating the mother centriole within a centrosome at the mitotic spindle pole. The *de novo* pathway remained inactive in the daughter cell that received the centrosome containing the ablated mother centriole, indicating that even an immature centriole is sufficient to maintain the *de novo* pathway fully suppressed. During the next mitosis the daughter containing the centrosome with the ablated mother centriole enacted bipolar spindles. Since only one of the spindle poles had a centriole-containing centrosome, one of the daughters of this cell received a normal diplosomal centrosome, while the sister receiving no centrosome, promptly proceeded to assemble centrosomes *de novo* upon reaching S phase (290). It is important to note that HeLa cells, as many other cancer cells, lack active p53 dependent checkpoint, and that this may enable progression through G1 in the absence of centrosomes. The *de novo* centrosome assembly pathway may therefore be not only an important back-up mechanisms in cancer cells to regenerate lost centrosomes, but also an intrinsically destabilizing process that leads to multipolar spindles and its attendant complications, if accidentally activated.

A detailed analysis of centrosome biogenesis in HU-arrested CHO (which are p53+/−), which lack a p53 dependent G1 checkpoint (293), has added new layers of complexity and indicated that the overreplication and *de novo* pathways may not fundamentally differ after all (311). Time-lapse imaging of centrin1-GFP expressing CHO cells revealed that perinuclear centrin spots appeared soon after HU arrest. These spot are quite similar to those appearing shortly after laser ablation of the centrosome, which are known to represent the earliest step in *de novo* centriole formation (290). Further characterization revealed that such structures correspond indeed to centriolar satellites, which are known to participate in centriologenesis. Such precursors could be traced to their formation in the nucleolus, export to the cytoplasm, coalescence round the native centrosome, development of centrioles, and acquisition of functional PCM (311). These observations suggest that the phenomenon of centrosome amplification commences through a dynein/dinactin-mediated buildup of PCM material in preparation for centrosome construction. Centriolar satellites are pericentriolar protein-rich, electron dense ∼100 nm quasi-spherical bodies that appear to represent assembly factories for centrosome or ciliary components (312–314). Payload traffic to and from centriole satellites is microtubule- and dynein/dynactin-dependent (311, 313–316).

A similar process has been reported by Kramer and collaborators using a lung cancer cell line carrying a centrin-2-Dendra2 transgene in which the fluorescent protein tag is photoconvertible from green to red, permitting to distinguish unambiguously between pre-existing centrioles (photoconverted red) from newly developed centrioles (green) (317). Using this system, again numerous centrin containing centriole satellites formed after gamma-irradiation or bleomycin exposure, before centrosome amplification became evident (317). In fact, all manners of DNA damage induced the appearance of green Centrin-2 dots, i.e., newly formed centrioles, with no instance in which splitting of red signals (i.e., pre-existing centrosomes) occurred, even after X-ray irradiation, where “centrosome splitting” was first described (317). Of note, the newly formed centrin dots were mobile and loosely associated with the pre-existing centrosome (red dots). When interrogated by immunofluorescence, the newly formed green dots were negative for canonical centrosome components such as pericentrin, γ-tubulin, C-Nap1, rootletin, SAS-6, and STIL, but positive for known components of centriolar satellites such as PCM-1, BBS-4, and CEP290. The resemblance of centrin dots to normal centriolar satellites extended to their ultrastructural appearance when examined by electron microscopy using nanogold-conjugated antibodies as tracers, indicating they represent excessive production of centriolar satellites. The appearance of centrin dots preceded the appearance of newly formed centrosomes. As expected for a centriolar satellite driven process, chemical inhibition of dynein or interference of dynein/dynacting function by overexpression of dynamitin, suppressed centrosome amplification induced by DNA damage or X-ray irradiation. Moreover, chemical inhibition of Chk-1 with UCN-01 led to dose-dependent reduction of centrin dots in A549 and U2OS cells. Similar results were obtained by siRNA knock-down of Chk-1 (317).

Unless resident centrosomes are non-functional (inactivated) in transformed/cancer cells arrested in S or G2 phase, the above two studies suggest that the *de novo* pathway is active in cancer cells despite the presence of resident centrosomes. Indeed, some observations suggest that not all centrosomes may be functional in cancer cells, as documented by free centrosomes not associated to spindle poles in some cancer cell lines (318). Moreover, it is formally possible that the Cent2 green dots observed in Loffler et al. study (317) are templated by resident centrioles. Recent observations provide an intriguing possible explanation to this puzzle (319). Treatment of cells with Cdk1 inhibitors (RO-3306, roscovitine) or Cdk1 knockdown with siRNA, results in G2 arrest, premature centriole disengagement and chromosome endoreduplication. Under these conditions premature centriole disengagement is dependent on both separase and Plk1 activation. It is well established that centriole disengagement and displacement is a pre-requisite for growth of new procentrioles and that a mitotic Plk1 activity is required to render a new centriole competent for procentriole nucleation, thus preventing the growth of “granddaughter” procentrioles/centrioles. Under these conditions separase activation obeys to destruction of securin by activated APC/C, mediated by Plk1 induced loss of early mitotic inhibitor 1 (Emi1) (320), which normally inhibits the APC/C. Subsequent inactivation of APC/C upon prolonged arrest triggers reaccumulation of cyclin A (and to some extent cyclin E) and increased cyclin A-Cdk2 activity, promoting centriole reduplication and DNA endoreduplication (319). Similar observations were made in CHO and U2OS HU-arrested cells suggesting that this response, which includes oscillation of APC/C activity leading to first centriole disengagement (high APC/C activity) and subsequent replication (low APC/C) may be universal to prolonged S or G2 arrests (319).

Although numerical abnormalities of centrosomes are the most common centrosome “cancer phenotype,” qualitative changes frequently co-occur. Qualitative changes are far less well characterized, and little is know about their impact on the fidelity of chromosome segregation during mitosis. They include abnormally shaped centrioles, excess or deficits in PCM, and acentriolar MTOCs (250, 251, 257, 270, 318, 321–325). With the recent advances in microscopy it is formally possible to study this phenomena *in vivo* by multiplexed high-resolution fluorescence microscopy. Such studies will provide a wealth of new information, rapidly identifying the critical molecular events, which implicitly are targets for tumor specific targeted therapies.

## Mitosis with Too Many Centrosomes: Spindle Multipolarity and Rectification Mechanisms

**Figure 5:**
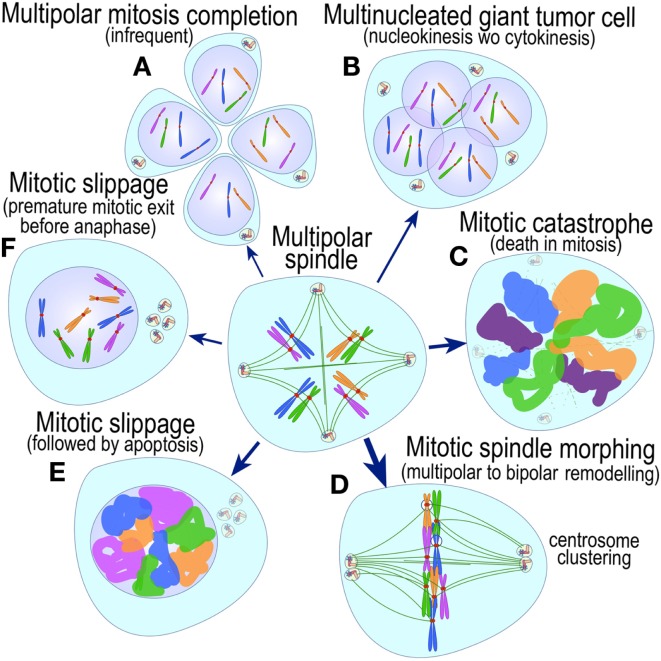
**Possible outcomes of multipolar mitoses in cancer**. A subset of cells with multipolar spindles carry mitosis to completion, resulting in highly aneuploidy cells, some with abnormal centrosome number **(A)**. Others fail cytokinesis resulting in giant multinucleated polyploid cells, often with supernumerary centrosomes **(B)**. Some cells exit mitosis in a process termed “mitotic slippage” and become polyploid cells with supernumerary centrosomes **(F)**, or apoptose in the subsequent G1 phase **(E)**. Yet others undergo mitotic catastrophe (death in mitosis) **(C)**. Finally, most cells with multipolar mitosis, after significant delay, reconfigure their multipolar spindles into bipolar spindles resulting in (mostly) normal or abnormal (merothelic, synthelic chromosome) chromosome segregation **(D)**. The thickness of the arrows in the figure intends to provide an estimate of the frequency of these events in cancer cells.

Regardless of the mechanism of origin, supernumerary centrosomes pose the same initial challenge to dividing cells: once two or more functionally mature centrosomes are present at the G2 phase of the cell cycle, the potential for multipolar spindles, and chromosome missegregation on the next mitosis is very real. However, the outcome of multipolar mitoses differs significantly depending on a number of additional factors (Figure 5). One key factor is the ploidy of the dividing cell, which influences the success rate of multipolar mitoses (see above). Additional factors include the ability of cancer cells with multipolar mitoses to circumvent the mitotic spindle assembly checkpoint (326); the competence of cell death execution pathways leading to mitotic (327) or post-mitotic cell death (328) of cells that cannot self-correct defects to satisfy the mitotic assembly checkpoint; the ability of the cell to exit mitosis without experiencing anaphase and cytokinesis – a process that has been termed “mitotic slippage”; and more importantly, the ability of the cell to reconfigure the multipolar spindle into a bipolar spindle before entering anaphase (328, 329) (Figure 5).

Mitoses with multipolar spindles are inherently inefficient, exhibiting a high rate of intra-mitotic (mitotic catastrophe) (327, 330, 331), post-mitotic cell death (328), or senescence (298), hindering tumor growth and acting as tumor suppressors rather than tumor promoters (332–334) (Figure 5). Multipolar cells that do undergo multipolar anaphase, but do not complete mitosis, and still survive, are likely to become giant multinucleated tumor cells (Figure 5). A subset of giant tumor cells is commonly present in cytologically high-grade tumors, but appear to either not divide at all or divide only sparingly. Rarely, cell with multipolar spindles may divide asymmetrically to produce viable daughter cells, a phenomenon that has been documented but does not appear to be prevalent, since the chances of a daughter cell receiving a full haploid chromosome complement is low. Nevertheless, the importance of such rare events should not be underestimated insofar as they may be critical in generating cells with properties significantly different from the main tumor population, which underscores, at least partly, the typical punctual evolution of the tumor genome (335–339).

**Figure 6:**
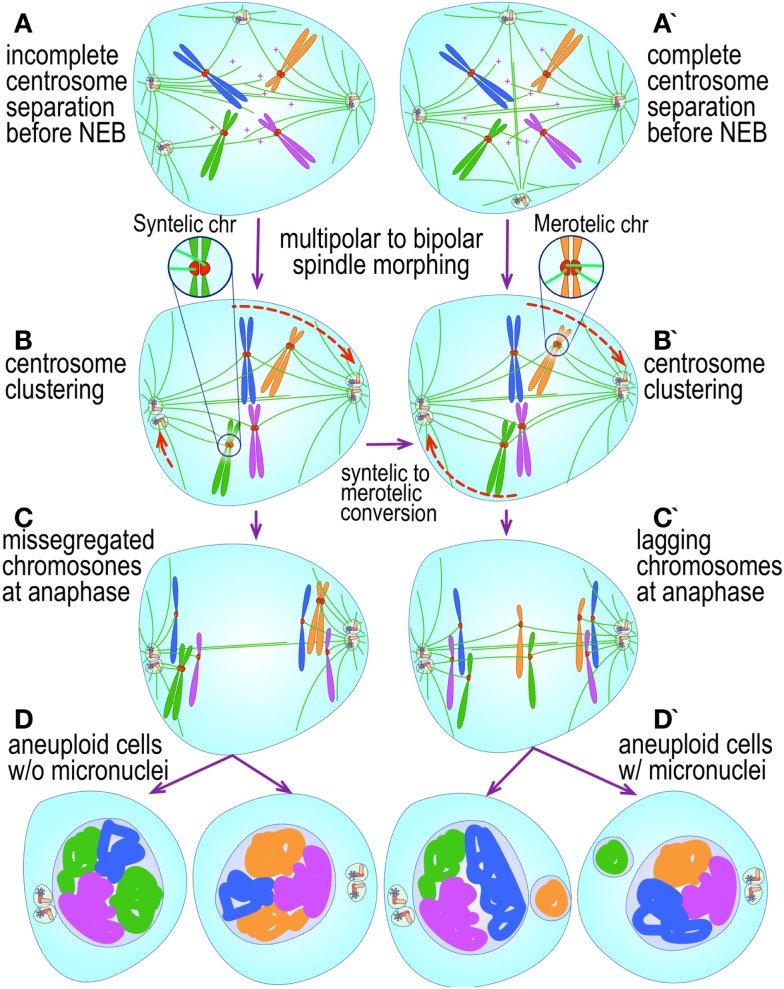
**Multipolar to bipolar spindle reconfiguration in cancer cells**. Cancer cells with supernumerary centrosomes enact multipolar spindles **(A,A’)**, but after some delay, reconfigure their spindles to bipolar structures with centrosomes clustered at the poles **(B,B’)**. Abnormal chromosome attachments acquired during the multipolar stage, such has synthelic **(B)** or merothelic **(B’)**, may then lead to monopolar segregation **(C)** or lagging **(C’)** chromosomes, which are usually incorporated into micronuclei, resulting in aneuploid cells with supernumerary centrosomes without **(D)** or with micronuclei**(D’)**.

Since a majority of multipolar mitosis outcomes are detrimental to cell growth (Figure 5) how might a tumor with supernumerary centrosomes prosper? How do cells with multipolar spindles solve the mitotic conundrum? Recent long-term *in vivo* observations of mitoses with multipolar spindles have revealed important clues. It turns out that a large proportion of cells with multipolar spindles utilize a spindle correction mechanism active in normal cells (340, 341) (Figure 6). After initially deploying multipolar spindles, cells delay metaphase until the extra spindle poles coalesce to form bipolar spindles, promoting “normal” bipolar mitoses (332, 341, 342). Multiple centrosomes per spindle poles were first observed by a number of investigators in mouse neuroblastoma cells (321, 323, 325), but their significance remained enigmatic until Bill Brinkley inferred their potential importance and predicted what indeed has been born out by recent experimental evidence (329). Centrosome coalescence in normal cells and some cancer cell lines is highly efficient (332, 343, 344) with cells rarely exhibiting multipolar mitoses even in the presence of greatly increased number of centrosomes (257). But not all cancers retain intact the centrosome clustering mechanisms and in some transformed cell lines (332, 341, 343, 344) and cancer tissue, multipolar anaphases may still occur. The extent to which multipolar mitoses contribute to CIN has not yet been determined. What mediates the metaphase delay needed to reconfigure a multipolar into bipolar spindle is at present poorly understood. The mitotic spindle assembly checkpoint (345) does not appear to monitor the number of spindle poles (346). However, it is possible that unattached or misattached kinetochores in multipolar spindles fail to generate the tensile forces that are key to extinguish the spindle assembly checkpoint (347, 348), delaying anaphase onset. Time spent in metaphase however is important since reducing it experimentally reduces centrosome clustering and leads to multipolar anaphases (340, 345, 349). The forces that contribute to centrosome clustering appear to be threefold: inter-centrosomal interactions, interactions of anti-parallel polar microtubules, and forces exerted by astral microtubules. Nevertheless, the molecular mechanisms coordinating these processes in cancer cells are poorly understood.

The correction mechanism itself seems to be dependent on kinetochore microtubule dynamics and interaction of polar microtubule bundles. The minus-end-directed microtubule motors nuclear mitotic apparatus protein (NuMA) and dynein have been implicated in this process (341). Two recent screens have implicated a number of other proteins in the process suggesting mechanistic possibilities. The first, a screen in non-transformed cells (*Drosophila* S2 cells) revealed three main classes of proteins: components of the mitotic spindle assembly checkpoint, regulators of cortical acto-myosin contractility, and microtubule associated proteins (MAPs) (340). Also discovered in the screen was an essential role in centrosome clustering for the non-essential microtubule minus-end-directed motor non-claret disjunctional (NCD), a kinesin-14 family member (vertebrate homolog HSET). Notably, HSET is not only critical for the formation of bipolar spindles in the absence of centrosomes – through the incorporation and clustering of MTOCs at spindle poles (318), but is also critical for the clustering of canonical centrosomes in cancer cell lines, but not in non-transformed RPE1 cells (318). A second genome-wide screen for proteins that participate in centrosome clustering, this time in tumor cells, identified proteins involved in kinetochore microtubule attachment, sister chromatid cohesion, members of the augmin complex microtubule formation pathway, and chromosome passenger complexes (CPC: aurora-B, INCENP, survivin, and borealin) (350). The study suggested that kinetochore and spindle components generate the forces necessary to maintain centrosome clustering at the poles.

## The Fate of Bipolar Mitoses with Clustered Centrosomes at Spindle Poles

Nevertheless, not all is well in cells that manage to convert multipolar to bipolar spindles. Multipolarity, even if transient (342), may lead to maloriented kinetochores, permitting microtubules from two or more poles to contact and bind the same kinetochore, leading to multipolar chromosome attachment (Figure 6). Upon resolution of multipolarity, the resulting bipolar spindle will contain merothelic or synthelic chromosomes. Merothelic attachments lead to lagging chromosomes at anaphase (332), resulting in anaphase bridges that interfere and delay cytokinesis. If the bridge is resolved and cytokinesis completed, the lagging chromosome becomes a micronucleus in one of the daughter cells. Alternatively, if the bridge is not resolved, the outcome is cytokinesis failure and polyploidy (Figure 6). Monothelic or synthelic attachments, which may also originate in multipolar spindles lead to monopolar segregation (both chromatids to the same daughter). Remarkably, cells with intact spindle assembly checkpoint may still correct some of these misattachments – whether merothelic, synthelic or monothelic – into perfectly amphitelic (bipolar) orientated chromosomes before anaphase onset, resulting in normal chromosome segregation (340–342).

## Not all Forms of Aneuploidy are Caused by Centrosome Abnormalities

Two forms of cancer aneuploidy are readily distinguishable in clinical cancer karyotypes: stable aneuploidy and unstable or dynamic aneuploidy. In the former, all cells in a cancer growth share gains and/or losses of the same normal or structurally abnormal chromosome(s), whereas in the latter, cancer cells have more extensive gains and losses of chromosomes, only some of which are shared by most of the cells in the tumor, while others are shared only by subsets of cells. Whereas the former is thought to results from rare and transient mitotic chromosome missegregation events in a founder cancer cell, the latter is due to frequent and continuous mitotic chromosome missegregation and is a symptom of an intrinsically defective chromosome segregation machinery (278). Unstable aneuploidy, which is also known as CIN (251, 351), is the more common of the two, and is pervasive in carcinoma, some forms of sarcoma, and a subset of hematopoietic and lymphoid cancers (251, 352) [reviewed in Ref. (16, 353)]. CIN is multifactorial and may result not only from centrosome dysfunction (250, 251), but also from defects in kinetochore microtubule attachment and dynamics (354, 355), spindle assembly checkpoint (356, 357), chromosome replication/condensation/cohesion (358), cytokinesis failure (291, 359, 360), or dysfunction of checkpoints that coordinate the DNA replication, and centrosome cycles (361).

## Centrosome Abnormalities Provide a Potential Mechanistic Link between Numerical and Structural Chromosome Abnormalities

**Figure 7:**
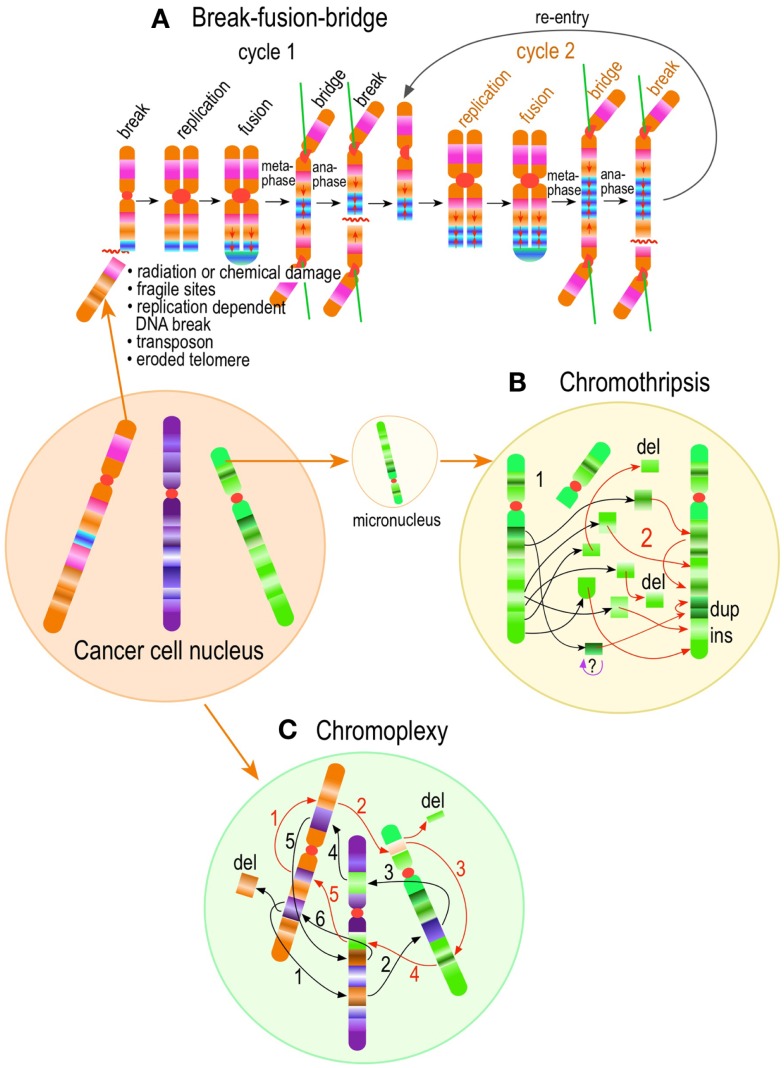
**Chromoanagenesis in cancer**. Complex structural chromosome rearrangements in cancer, involve stepwise or punctual chromosome restructuring. It includes stepwise classic intra or inter-chromosomal break-fusion-bridge cycles **(A)**; chromothripsis **(B)**, which are punctual localized highly complex chromosome fragmentation (1) and rejoining (2) events, and chromoplexy **(C)**, in which complex linked translocation events involving multiple chromosomes presumably occur simultaneously. 1 through 6 (black typeface) and 1 through 5 (orange typeface) represent two such linked “chained” events.

In CIN, numerical (nCIN) and structural (sCIN) chromosome abnormalities nearly always co-exist [reviewed in Ref. (362–365)]. nCIN includes a spectrum of gain and losses of chromosomes fragments from kilobases to megabases, whole arms or even entire chromosomes. sCIN include translocations, inversions, end-fusions, and a number of more complex rearrangements. With the exception of break-fusion-bridge (BFB) cycles (366) (Figure 7), until recently it was thought that nCIN and sCIN, despite their frequent coexistence, were largely mechanistically unrelated. Several recent whole-genome sequencing studies of cancer tissue have uncovered new mechanistic links between nCIN, sCIN, and centrosomes (Figure 7). Grouped under the term chromoanagenesis (chromosome rebirth) (367), the first class of sCIN consists of a handful of apparently random chromosome loci per genome with highly complex structural (and copy number) sequence alterations including sequence duplications, deletions, scrambling, and polarity reversals, as if the segment had been broken in hundreds of fragments and rejoined more or less randomly (Figure 7). This phenomenon has been termed chromothripsis (chromosome shattering) (368). Chromothripsis has been postulated to occur as a single, punctual, massive event, rather than sequentially, as is seen with BFB cycles. Two possible causative mechanisms have been thus far delineated, both of which are enabled by chromosome missegregation events triggered by abnormal centrosome function in mitosis. Chromothripsis involves premature mitotic entry of a chromosome contained in a micronucleus that resulted from a chromosome missegregation event in the previous mitosis (368, 369). Mitotic entry, before completion of DNA replication in the micronucleus, leads to failure of micronucleus envelope breakdown, mitotic transit with random segregation to daughter cells, and random reassembly of the incompletely replicated chromosome fragments within the micronucleus during the subsequent interphase (368, 369). Chromothripsis [reviewed in (362, 363, 370)] appears to be common in carcinoma and neural tumors (369, 371) and is the mechanism most likely to operate in micronuclei. Micronuclei in cancer are frequent and may have many origins, one of the most important of which appears to be merotelic chromosome orientation at metaphase. A merothelic chromosome is one in which one of its two kinetochores is simultaneously attached by microtubules to the two spindle poles. Merothelic chromosomes often lag at the center of the dividing cell at anaphase and are usually not incorporated within the two groups of chromosomes at the poles in telophase before reassembly of the nuclear envelope at the end of mitosis, becoming independent “micronuclei.” Notably, a transient state of mitotic spindle multipolarity in cells with supernumerary centrosomes is thought to be the most important cause of merothelic chromosome attachment and micronuclei formation (355) (*vide infra*), again implicating centrosome dysfunction in the cause of sCIN.

A second class of distinct “genome level” structural abnormality in cancer gleaned from whole-genome sequences has been recently delineated in prostate cancer (335). Termed chromoplexy (chromosome restructuring), in this phenomenon numerous inter- and intra-chromosome translocations and deletion of genetic material arise in a highly interdependent manner (335) (Figure 7). These “chained rearrangements,” numbering from 3 to over 40, involved up to 6 chromosomes simultaneously, exhibited precise joints or large deletions at the joints, and occurred in the majority of prostate cancers studied. Moreover, greater than 60% of the tumors contained more than one chained rearrangements (335, 339). Statistical analyses indicated these rearrangements were unlikely to occur independently, suggesting again a single, punctual, massive genome-scrambling event (339). The cause(s) and mechanism(s) of chromoplexy, and whether centrosomes participate in its pathogenesis, are currently unknown.

## Summary

A significant body of evidence implicating centrosome dysfunction on CIN has accumulated in the past 20 years. Centrosome defects are ubiquitous in cancer and are associated with dynamic CIN due to chromosome missegregation during mitosis. Surprisingly, centrosome dysfunction also participates in promoting structural CIN by a number of mechanisms, principally by initiating micronucleus formation through merothelic chromosome attachments, chromosome breakage at centromeres, and DNA damage on miss attached chromosome. The combination of structural and numerical chromosome abnormalities triggered by centrosome dysfunction, ultimately leads to gene reshuffling and reprograming of the cancer genome. Reprograming results primarily from four related events associated with gene reshuffling: deregulation of gene expression resulting from gene repositioning, which changes both the regulatory element landscape and chromatin regulatory domains; gene dosage changes resulting from numerical changes of part or whole chromosomes; gene mutations (gene fusion, inversions, indels, etc.) resulting from chromosome domain repositioning; and changes in the feasibility and probability of oncogene gain or tumor suppressor gene loss by dissociating genes from neighboring genes that normally exert opposite selection pressure. Importantly, cancer cells have developed mechanisms to cope with altered centrosome function, primarily by reconfiguring multipolar spindles into bipolar spindles prior to anaphase. The intricate molecular defects of centrosome dysfunction in cancer provide a unique opportunity for hyper-targeted therapies, which not only interfere with a specific molecule also present in normal cells, but also with a process specifically deranged in cancer, on principle avoiding harm to normal cells. Technical advances in microscopy, fluorescent protein technology and high throughput screening will permit a more rigorous examination of centrosome defects and their functional consequences in short-term cultures of human cancer samples and immortalized “non-transformed” human cells. Only through this exercise will we fully understand the magnitude and the critical differences in centrosome biology between normal and cancer tissue permitting us to develop smart therapies to combat “the emperor of all maladies.”

## Conflict of Interest Statement

The author declares that the research was conducted in the absence of any commercial or financial relationships that could be construed as a potential conflict of interest.
